# Pulmonary complement depositions in autopsy of critically ill patients have no relation with ARDS

**DOI:** 10.1186/s40635-019-0237-2

**Published:** 2019-07-25

**Authors:** Friso M. de Beer, Mark P. V. Begieneman, Joris J. T. H. Roelofs, Janneke Horn, Hans W. M. Niessen, Marcus J. Schultz, Wim K. Lagrand

**Affiliations:** 1 Laboratory of Experimental Intensive Care and Anesthesiology (L·E·I·C·A), Amsterdam UMC, University of Amsterdam, Amsterdam, The Netherlands; 2 Department of Intensive Care, Amsterdam UMC, University of Amsterdam, Amsterdam, The Netherlands; 3 Department of Anesthesiology, Amsterdam University Medical Centers, Academic Medical Center, Room H1-118, Meibergdreef 9, 1105AZ Amsterdam, The Netherlands; 4 Department of Pathology, Netherlands Forensic Institute, Den Haag, The Netherlands; 5 Department of Pathology, Amsterdam UMC, University of Amsterdam, Amsterdam, The Netherlands; 6 Department of Pathology, Amsterdam UMC, Vrije Universiteit Amsterdam, Amsterdam, The Netherlands; 7 Mahidol Oxford Tropical Medicine Research Unit (MORU), Mahidol University, Bangkok, Thailand

**Keywords:** Intensive care, ARDS, Autopsy, Complement, Complement activation, Complement deposition, C3d, Diffuse alveolar damage, Lung injury score, Driving pressure

## Abstract

**Background:**

The complement system has frequently been suggested to play a role in the pathophysiology of acute respiratory distress syndrome (ARDS). The current study explored the association between pulmonary depositions of a complement activation product and the clinical diagnosis of ARDS.

**Methods:**

Lung tissue material from autopsied critically ill patients who died whilst on invasively mechanical ventilation was collected and stained for complement C3d. The diagnosis of ARDS was by the Berlin Definition. Lung injury scores (LIS) and driving pressures were calculated, 48 and 24 h prior to death. A pathologist who remained blinded for the clinical data scored the extent of C3d depositions, using a C3d deposition score (a minimum and maximum score of 0 and 24), and of diffuse alveolar damage (DAD). The primary analysis focused on the association between the C3d deposition score and the clinical diagnosis of ARDS. Secondary analyses focused on associations between the C3d deposition score and the presence of diffuse alveolar damage (DAD) in histopathology, and LIS and driving pressures in the last 2 days before death.

**Results:**

Of 36 patients of whom autopsy material was available, 12 were diagnosed as having had ARDS. In all patients, C3d depositions were found in various parts of the lungs, and to a different extent. Notably, C3d deposition scores were similar for patients with ARDS and those without ARDS (4.5 [3.3–6.8] vs. 5.0 [4.0–6.0]; *not significant*). C3d deposition scores were also independent from the presence or absence of DAD, and correlations between C3d scores and LIS and driving pressures prior to death were poor.

**Conclusion:**

Pulmonary C3d depositions are found in the lungs of all deceased ICU patients, independent of the diagnosis of ARDS. The presence of complement C3d was not associated with the presence of DAD on histopathology and had a poor correlation with ventilation characteristics prior to death.

## Background

Acute respiratory distress syndrome (ARDS), a frequent complication of critical illness, is associated with high mortality and morbidity [1, 2]. A specific therapy is presently lacking. Preclinical studies using various models of lung injury [3–6], as well as human investigations in intensive care unit (ICU) patients with ARDS, have shown complement activation in the lung [7, 8]. It is uncertain, though, whether this finding is unique for ARDS and also whether the extent of complement activation depends on ARDS severity and ventilator settings at the moment of sampling.

The current study explored the presence and extent of pulmonary depositions of a complement activation product, C3d, and its association with the clinical diagnosis of ARDS. This study also explored whether pulmonary C3d deposition is associated with the presence of diffuse alveolar damage (DAD) on histopathology, a characteristic finding of ARDS [9], and the lung injury score (LIS) [10] and driving pressures in the last days before death. For this, autopsy material of critically ill patients who received invasive mechanical ventilation till death was collected, immunohistochemically stained, and analyzed.

## Methods

### Patients

In the Amsterdam University Medical Center, location Academic Medical Center (AMC), it is a routine practice to ask consent from family members of deceased patients for an autopsy. In up to 20% of cases, consent to perform an autopsy is obtained. Consent includes permission to store autopsy material for research at a later moment. Dutch law does not require reconsent.

### Inclusion and exclusion criteria

The ICU registry and autopsy registry of the department of pathology were merged to identify patients who died in the ICU during a 3-year period. Patients were included if a clinical autopsy was performed and when autopsy material was stored and still available. This study had no exclusion criteria.

### Clinical data and ventilation parameters

Baseline demographic data, including age, gender, body mass index (BMI), acute physiology and chronic health evaluation (APACHE) IV scores, Simplified Acute Physiology Scores (SAPS), and reasons for ICU admission, were retrospectively collected from the patient data management system (MetaVision®, iMDsoft, Tel Aviv, Israel).

Hourly validated ventilator parameters, including fraction of inspired oxygen (FiO_2_) levels, positive end-expiratory pressure (PEEP), maximum inspiratory pressure (Pmax), and tidal volume, were collected, as well as arterial blood gas analysis results and all plain chest radiography films. Daily medians of ventilator parameters were calculated in order to compare data between patients.

### Clinical diagnosis of ARDS

In retrospect, it was determined whether patients had ARDS, and its severity, using the Berlin Definition [11]. For this, all available chest radiographs were re-examined, a partial arterial oxygen pressure (PaO_2_) to FiO_2_ ratios were re-calculated, and the medical history was sought for risk factors for ARDS. The Berlin Definition was strictly used. Therefore, patients must have had a respiratory failure within 1 week of a known clinical insult; have had bilateral opacities that were not fully explained by, e.g., effusions, or lung collapse; and respiratory failure was not fully explained by cardiac failure or fluid overload. For calculation of the classifying PaO_2_/FiO_2_, patients must have received at least 5 cm H_2_O PEEP at the moment of their collection. For determination of ARDS severity, the worst matched PaO_2_ and FiO_2_ was used. Finally, the investigators had to reach retrospective consensus in all patients.

### LIS and driving pressure

To calculate the LIS [10] and driving pressure, data of the last 48 h before death were used. Driving pressure was calculated at 48 and 24 h before death by subtracting PEEP from Pmax.

### Tissue handling and immunohistochemical staining

With each autopsy within the AMC, it was policy to obtain and store macroscopically afflicted lung tissue. The tissue was originally fixed in 4% formalin and embedded in paraffin.

Lung tissue captions were stained with hematoxylin and eosin or immunohistochemically stained for complement factor C3d. For this, 4-μm sections were dewaxed, rehydrated, and incubated in methanol/H_2_O_2_ (0.3%) for 30 min to block endogenous peroxidases. Next, antigen retrieval was performed by heat inactivation in citrate buffer (pH 6.0) for slides to be stained for complement C3d. Then, slices were incubated with rabbit anti-human complement C3d (1:1000) (Dako Cytomation, Eindhoven, The Netherlands) for 1 h at room temperature. Next, sections were incubated with Envision (undiluted, anti-mouse/anti-rabbit, Dako Cytomation) for 30 min at room temperature. Staining was visualized using 3,3′-diaminobenzidine (DAB, 0.1 mg/ml, 0.02% H_2_O_2_). Sections were subsequently counterstained with hematoxylin, dehydrated, and covered. With each staining, a phosphate-buffered saline (PBS) control and an isotype control was included. PBS and isotype control staining yielded negative results in all cases, suggesting specific antibody binding.

### Pathohistological examination and scoring

One experienced pathologist (J.J.T.H.R.) who remained blinded to patient group allocation (ARDS vs. non-ARDS) assessed C3d depositions in the lung tissue. For quantification of C3d deposition in the lung, C3d staining intensity was assessed in the following tissue components: endothelium, vascular intima, arterial lamina elastic, pneumocytes, bronchial epithelium, macrophages, and hyaline membranes. In addition, staining for C3d was assessed in areas of pulmonary edema. Each variable was graded on a scale of 0 to 3 for C3d deposition intensity, as follows: 0, absent; 1, slightly present; 2, moderate present; and 3, intensively present [12]. The sum of scores for all variables was the complement deposition score resulting in a score range between 0 and 24.

The same pathologist scored the presence of DAD on hematoxylin and eosin stained slices. The presence of DAD mandated the presence of the hyaline membranes plus at least one of the following: intra-alveolar edema, alveolar type I cell necrosis, alveolar type II cell proliferation, the interstitial proliferation of fibroblasts and myofibroblasts, or organizing interstitial fibrosis [9].

### Endpoints

The primary endpoint was the association between the pulmonary C3d deposition score and the clinical diagnosis of ARDS. Secondary endpoints were the association between extent of C3d deposition and the presence of DAD, and the correlation between C3d deposition and LIS and driving pressure (both at 48 and 24 h prior to death).

### Statistical analysis

Data were presented as mean with standard deviation (SD) or median with interquartile range [IQR], where appropriate. Categorical variables were expressed as *n* (%). Baseline characteristics between patients with and without ARDS were compared using Student’s *t* test or Mann-Whitney *U* test according to data distribution or Fisher’s test comparing with categorical variables.

For the correlation between the complement deposition score and DAD, the LIS and driving pressure, the correlation coefficient (*r*) using Pearson’s/Spearman/nonlinear association method according to data distribution.

Statistical analysis was performed using GraphPad Prism version 7.01 (GraphPad Software Inc., La Jolla, CA, USA). A *P* value of < 0.05 was considered statistically significant.

## Results

### Patients

**Fig. 1:**
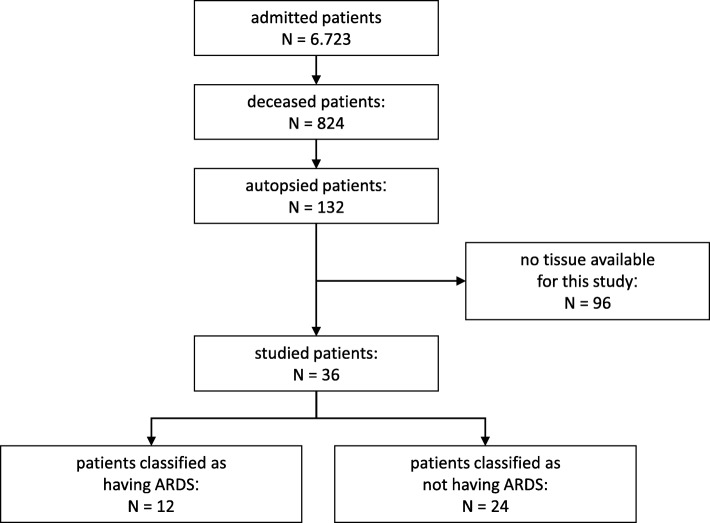
Flow of patients and availability of autopsy material

Patient flow is presented in Fig. 1. Of all admitted patients, 12% died in ICU. Of them, 16% were autopsied. The autopsy tissue was available for only 36 patients. Of them, 12 patients fulfilled the Berlin Definition criteria for ARDS; one patient was classified as having mild ARDS, and four and seven as moderate or severe ARDS, respectively. Six patients (50%) had ARDS at admission and the number of days from ARDS onset to death was 4.0 [2.0–15.0]. In ARDS patients, risk factors for ARDS were pneumonia (*n* = 7), sepsis (*n* = 4), and pancreatitis (*n* = 1). In patients without ARDS, risk factors were pneumonia (*n* = 5), sepsis (*n* = 5), severe burns (*n* = 1), and major trauma (*n* = 1). Pneumonia was significantly more present in ARDS patients. DAD was present in 9 ARDS patients (75%) who fulfilled the Berlin Definition for ARDS, but also in 14 patients (58%) without ARDS. Patients with DAD without ARDS had variable underlying pathologies. Four patients suffered septic shock with multi-organ failures. The residual patients suffered in-hospital cardiac arrest (*n* = 2), cardiogenic shock with multi-organ failures (*n* = 2), cardiogenic and hypovolemic shock with multi-organ failures (*n* = 2), acute pancreatitis and liver cirrhosis (*n* = 1), multi-trauma (*n* = 1), exacerbation of pulmonary fibrosis based on sarcoidosis (*n* = 1), and cerebral hemorrhage and airway aspiration (*n* = 1).

Patient demographics and ventilation characteristics are presented in Tables 1 and 2. No difference in duration of mechanical ventilation was found between patients with or without ARDS (4.5 [2.8–15.0] vs. 4.5 [2.0–13.3] days; *NS*). The LIS was higher in ARDS patients compared to non-ARDS patients, both at 48 and at 24 h prior to death. The driving pressure was different between ARDS patients and non-ARDS patients at neither time point.

**Table 1:** Patient characteristics

ARDS vs non-ARDS	ARDS	Non-ARDS	*P* value
	*N* = 12	*N* = 24	
Age in years, mean ± SD	56.2 ± 13.7	68.0 ± 11.4	0.017
Female gender, *n* (%)	3 (25.0)	8 (33.3)	0.609
BMI (kg/m^2^), mean ± SD	23.9 ± 6.2	27.8 ± 5.1	0.054
Pulmonary history, *n* (%)	3 (25.0)	7 (29.2)	0.793
Admission type			
Medical, *n* (%)	8 (66.7)	13 (54.2)	0.473
Surgical emergency, *n* (%)	2 (16.7)	5 (20.8)	0.766
Surgical planned, *n* (%)	2 (16.7)	6 (25.0)	0.571
ARDS			
Mild, *n* (%)	1 (8.3)		
Moderate, *n* (%)	4 (33.3)		
Severe, *n* (%)	7 (58.3)		
Risk factors ARDS			
Pneumonia, *n* (%)	7 (58.3)	5 (20.8)	0.024
Sepsis, *n* (%)	4 (33.3)	5 (20.8)	0.414
Pancreatitis, *n* (%)	1 (8.3)	0 (0)	0.151
Severe burns, *n* (%)	0 (0)	1 (4.2)	0.473
Major trauma, *n* (%)	0 (0)	1 (4.2)	0.473
ARDS at admission, *n* (%)	6 (50)		
Days ARDS to death, median [IQR]	4.0 [2.0–15.0]		
APACHE IV score, mean ± SD	23.4 ± 6.4	26.1 ± 8.3	0.334
SAPS score, mean ± SD	56.7 ± 11.6	58.1 ± 17.5	0.802
LIS 48 h, mean ± SD	2.6 ± 0.8	1.8 ± 0.9	0.011
LIS 24 h, mean ± SD	2.8 ± 0.9	1.9 ± 0.9	0.008
MV days, median [IQR]	4.5 [2.8–15.0]	4.5 [2.0–13.3]	0.611
ICU length of stay in days, median [IQR]	7 [2.8–16.8]	9.5 [2.0–15.8]	0.809
DAD with hyaline membranes, *n* (%)	9 (75.0)	14 (58.3)	0.326
Complement deposition, median [IQR]	4.5 [3.3–6.8]	5.0 [4.0–6.0]	0.784

*BMI* body mass index, *ARDS* Acute Respiratory Distress Syndrome, *APACHE* Acute Physiology and Chronic Health Evaluation, *SAPS* Simplified Acute Physiology Score, *LIS* lung injury score, *DAD* diffuse alveolar damage

**Table 2:** Ventilation characteristics

ARDS vs non-ARDS	ARDS	Non-ARDS	*P* value
	*N* = 12	*N* = 24	
*48 h prior to death*			
Driving pressure, median [IQR]	17.0 [12.4–22.8]	13.0 [11.0–19.8]	0.160
PEEP, median [IQR]	13.5 [7.4–15.8]	7.0 [5.0–13.0]	0.048
Pulmonary compliance, median [IQR]	25.2 [19.8–41.2]	30.6 [23.2–43.9]	0.394
PaO_2_/FiO_2_ ratio, median [IQR]	160 [108–192]	190 [170–274]	0.031
Tidal volume IBW, median [IQR]	7.0 [5.9–8.1]	7.1 [6.3–7.8]	0.857
*24 h prior to death*			
Driving pressure, median [IQR]	18.8 [14.5–22.0]	17.0 [12.0–20.5]	0.210
PEEP, median [IQR]	13.5 [6.5–15.0]	5.0 [5.0–10.5]	0.031
Pulmonary compliance, median [IQR]	26.6 [19.1–33.9]	30.1 [25.4–41.7]	0.238
PaO_2_/FiO_2_ ratio, median [IQR]	116 [93–186]	189 [160–276]	0.010
Tidal volume IBW, median [IQR]	6.6 [5.9–8.2]	6.8 [6.4–8.1]	0.542

*PEEP* positive end-expiratory pressure, *PaO*_*2*_ partial arterial oxygen pressure, *FiO*_*2*_ fraction of inspired oxygen, *IBW* ideal body weight

### Pulmonary C3d depositions

C3d depositions were found in vascular intima, arterial lamina elastica, bronchial epithelium, macrophages, edematous areas, and hyaline membranes, but not on endothelium and pneumocytes. All patients, i.e., patients with ARDS as well as those without ARDS, had positive staining for complement C3d to some extent, in at least one or more of the abovementioned areas.

**Fig. 2:**
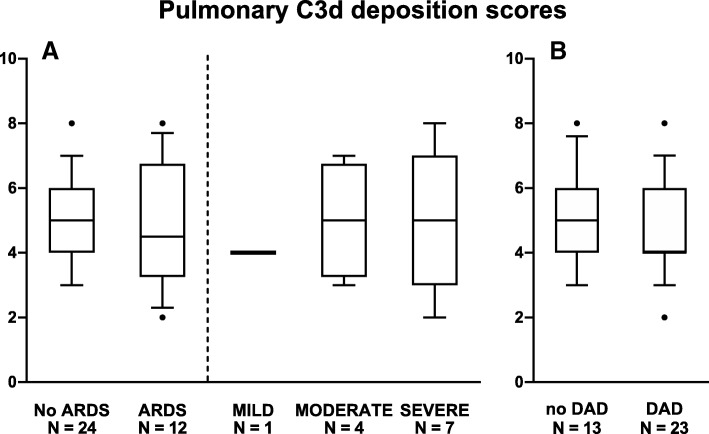
**a** Pulmonary C3d deposition scores in patients without ARDS compared to patients with ARDS and subdivided into ARDS severity. **b** C3d deposition in the lung tissue of patients with and without DAD. Boxplots and whiskers are 25–75% and 10–90%; the dots represent outliers

C3d deposition scores were not different between patients with ARDS and patients without ARDS (4.5 [3.3–6.8] vs. 5.0 [4.0–6.0]; *NS*) (Fig. 2a). Even when comparing the extremes, i.e., patients with severe ARDS and patients without ARDS, showed no differences in the C3d deposition scores. The extent of C3d deposition was independent from the time between ARDS onset, or the start of mechanical ventilation, and death (data not shown).

### Correlation between C3d depositions and the presence of DAD, LIS, and driving pressures

**Fig. 3:**
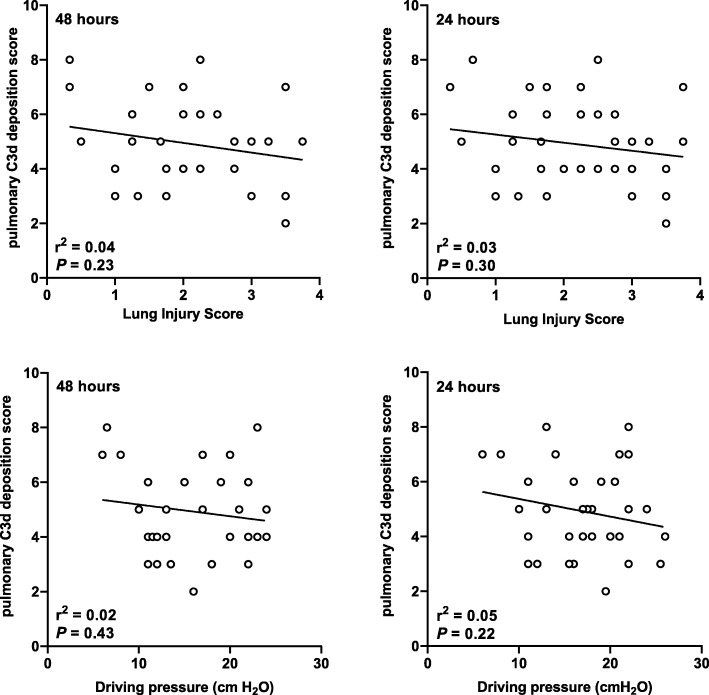
Correlations between pulmonary C3d deposition scores and lung injury scores and driving pressures at 48 and 24 h prior to death

The extent of C3d depositions was not different between patients who had no DAD and patients who had DAD in the lungs (5.0 [4.0–6.0] vs. 4.0 [4.0–6.0]; *NS*) (Fig. 2b). The extent of C3d depositions had a poor correlation with LIS and driving pressures in the last days before death (Fig. 3).

## Discussion

The findings of this autopsy study in critically ill patients who died when receiving invasive ventilation can be summarized as follows: (1) C3d deposition in the lung tissue was found in all patients, irrespective of the clinical diagnosis of ARDS; (2) pulmonary C3d deposition scores were not different between ARDS patients and those who did not fulfill the criteria for ARDS; and (3) did not increase with ARDS severity. Furthermore, (4) C3d deposition scores showed no association with the presence of DAD, and (5) the extent of C3d deposition had no correlation with the LIS and the driving pressure in the last days before dying.

One strength of the current study is that a strict definition for ARDS was used in retrospect, i.e., the diagnosis of ARDS was not based on the bed-side clinicians who may have missed the diagnosis or may have missed to report whether or not ARDS was clinically present. Another strength is that, apart from using the clinical diagnosis, also the presence of DAD was used to further classify the patients. Also, to our knowledge, quantifying complement deposition in the lung tissue has not been done before. The method using a cumulative score for different components of a (lung) tissue slide is a frequently used and well established for quantification of immunohistochemical staining [12, 13].

The findings of this study are remarkable, as they are contrasting findings of several preclinical studies in animals [3–6, 14] and two clinical investigations [7, 8]. Indeed, all these earlier studies showed an association between pulmonary complement activation and ARDS. There are several differences, however, between the current study and the preceding clinical studies that could explain, at least in part, the dissimilarities. First, in both preceding studies, bronchoalveolar lavage fluid was examined for complement activation [7, 8], while the lung tissue was used in the current investigation. Of note, ARDS is a disease of the lung tissue, and examination of the lung tissue, therefore, could better reflect pulmonary complement activation than the lung lavage fluid. Also, one of the preceding clinical investigations used healthy volunteers as controls [7], while critically ill patients without ARDS were used as a control group in the current investigation. It could be that complement activation in the previous investigation reflects an effect of critical illness, and maybe lung injury caused by mechanical ventilation, more than that it reflects ARDS. Remarkably, in that same study [7], ARDS patients with persistent hypoxia at high oxygen fractions were excluded from participation, possibly resulting in an underrepresentation of severe ARDS patients. Hereby, a possible underestimation of pulmonary complement activation in ARDS patients could have occurred in that study. Nevertheless, in the current study, the extent of pulmonary C3d depositions was similar in all patients irrespective of the severity of ARDS. The other clinical study showed increased C3a levels in bronchoalveolar lavage fluid within the first 12 h only and not thereafter [8]. In the current study, the time between onset of ARDS and death was highly variable, but in almost all cases longer than 12 h. Further analysis showed no association between the extent of C3d deposition and time between ARDS onset and death. Finally, in that same clinical study, the focus was entirely on ARDS from the traumatic origin, and ventilation data of the included patients were not reported [8].

In the absence of a specific therapy for ARDS, one option for treatment that remained open was inhibition of the complement system. One noticeable finding was that even in the extremes of respiratory failure, i.e., no ARDS versus severe ARDS, there were no differences in the pulmonary C3d deposition scores. The correlation with markers of disease severity at later time points, i.e., by focusing on the LIS and driving pressures in the last days before death, was also poor. While the present study cannot totally exclude a role for inhibition of the complement system, it certainly does not favor future investigations for this intervention in ARDS.

Several complement factors are potentially available to measure complement activation in plasma. Although complement anaphylatoxins (C3a, C4a, and C5a), as effector molecules of the complement system, are of interest in ARDS, these biomarkers are very unstable, soluble, with very short half-life time and are therefore not suitable for measurement in the post mortem tissue. In our study, we therefore used complement C3d in our post mortem tissue, as C3d has previously been proven to be an adequate tissue marker of complement activation in immunohistochemical staining studies [15].

Diffuse alveolar damage (DAD) has frequently been used as a hallmark sign for ARDS by pathologists. A recent post mortem study, however, showed that less than 50% of patients with clinical ARDS actually had DAD on autopsy [16]. Some argue that DAD could be more a resultant or a reflection of the harmful effects of ventilation. The use of low tidal volumes may result in less DAD. In the current study, all patients received lung-protective ventilation with low tidal volumes, which could explain, in part, why many patients with clinical ARDS showed no DAD on autopsy.

There was a high prevalence of DAD in non-ARDS patients in our study. However, the finding of DAD in non-ARDS patients is not novel. In previous studies, DAD was not solely found in patients with ARDS. In two previous autopsy studies, DAD was present in respectively 4.5 and 11.5% of the non-ARDS patients when no risk factors for ARDS were present. When non-ARDS patients had risk factors for ARDS, the presence of DAD increased to 7.6 and 39.5%, respectively [16, 17]. Also in a recent study for open lung biopsy (OLB) in ARDS, DAD was present in 26.7% of non-ARDS patients [18]. Furthermore, previous OLB research showed DAD not to be uncommon in unilateral lung disease [19].

It could be argued that time between death and tissue collection on autopsy may have been too long, as probably not all patients were autopsied within the same day of dying. Nevertheless, a study using human myocardial tissue suggest that tissue characteristics are well preserved in time [15], albeit that the conservation methods in that study differed from the method used here.

The current study had several limitations. This study was relatively small, with a limited number of patients with clinical ARDS. Also, only one lung tissue section per patient was examined, which poses a risk for missing parts of the disease process. However, during the clinical autopsy, samples were taken from macroscopically most afflicted lung areas. The way driving pressure was calculated could be criticized, since spontaneous breathing activity was unknown. An underestimation of the driving pressure might therefore have occurred.

## Conclusions

In conclusion, in contrast to findings from previous studies suggesting a pathophysiologic role for complement activation in ARDS, the results of this study show no differences in pulmonary C3d depositions between patients with ARDS and those without ARDS. This finding suggests no role for complement activation in ARDS.

## Abbreviations

APACHE: Acute physiology and chronic health evaluation
ARDS: Acute respiratory distress syndrome
BMI: Body mass index
DAB: 3,3′-Diaminobenzidine
DAD: Diffuse alveolar damage
FiO_2_: Fraction of inspired oxygen
ICU: Intensive care unit
LIS: Lung injury score
OLB: Open lung biopsy
PaO_2_: Partial arterial oxygen pressure
PBS: Phosphate-buffered saline
PEEP: Positive end-expiratory pressure
Pmax: Maximum inspiratory pressure
SAPS: Simplified Acute Physiology Scores
